# Combining Cell-Free Protein Synthesis and NMR Into a Tool to Study Capsid Assembly Modulation

**DOI:** 10.3389/fmolb.2019.00067

**Published:** 2019-08-08

**Authors:** Shishan Wang, Marie-Laure Fogeron, Maarten Schledorn, Marie Dujardin, Susanne Penzel, Dara Burdette, Jan Martin Berke, Michael Nassal, Lauriane Lecoq, Beat H. Meier, Anja Böckmann

**Affiliations:** ^1^Institut de Biologie et Chimie des Protéines, MMSB, Labex Ecofect, UMR 5086 CNRS, Université de Lyon, Lyon, France; ^2^Physical Chemistry, ETH Zurich, Zurich, Switzerland; ^3^Gilead Sciences, Foster, CA, United States; ^4^Janssen Pharmaceutica N.V., Beerse, Belgium; ^5^Internal Medicine II/Molecular Biology, University Hospital Freiburg, Freiburg, Germany

**Keywords:** cell-free protein synthesis, NMR, proton detection, capsid, HBV—hepatitis B virus, assembly modulation

## Abstract

Modulation of capsid assembly by small molecules has become a central concept in the fight against viral infection. Proper capsid assembly is crucial to form the high molecular weight structures that protect the viral genome and that, often in concert with the envelope, allow for cell entry and fusion. Atomic details underlying assembly modulation are generally studied using preassembled protein complexes, while the activity of assembly modulators during assembly remains largely open and poorly understood, as necessary tools are lacking. We here use the full-length hepatitis B virus (HBV) capsid protein (Cp183) as a model to present a combination of cell-free protein synthesis and solid-state NMR as an approach which shall open the possibility to produce and analyze the formation of higher-order complexes directly on exit from the ribosome. We demonstrate that assembled capsids can be synthesized in amounts sufficient for structural studies, and show that addition of assembly modulators to the cell-free reaction produces objects similar to those obtained by addition of the compounds to preformed Cp183 capsids. These results establish the cell-free system as a tool for the study of capsid assembly modulation directly after synthesis by the ribosome, and they open the perspective of assessing the impact of natural or synthetic compounds, or even enzymes that perform post-translational modifications, on capsids structures.

## Introduction

The hepatitis B virus (reviewed in Nassal, 2008; Seeger and Mason, 2015) is a small enveloped DNA virus whose genomic information encodes few genes: the envelope proteins S, M and L (collectively known as hepatitis B surface antigen/HBsAg), the core protein (Cp), the polymerase (P), and the X protein (HBx). The icosahedral HBV capsid is formed by Cp, the different functions of which are driven by phosphorylation/dephosphorylation of its C-terminal domain (Kann and Gerlich, 1994; Gazina et al., 2000; Blondot et al., 2016; Ludgate et al., 2016; Heger-Stevic et al., 2018b). Cp is a 183-residue protein with two domains: the assembly domain that forms the contiguous capsid shell, and the C-terminal domain (CTD, residues 150–183) that amongst other functions is responsible for RNA packaging (Birnbaum and Nassal, 1990). The two domains are connected by a linker (residues 141–149). In infected cells, the core proteins pack the pregenomic (pg) RNA on assembly (Nassal, 1992), as well as a copy of the viral polymerase (Bartenschlager et al., 1990). Inside the capsid, the pgRNA is then transcribed to double-stranded relaxed circular (rc) DNA, generating mature capsids ready for envelopment.

The core protein thus plays essential roles at different stages of the virus life cycle and currently emerges as a promising drug target (Zlotnick et al., 2015) (recently reviewed in Yang and Lu, 2018; Nijampatnam and Liotta, 2019), with development of corresponding, effective antiviral agents well under way. Molecules targeting Cp are often called capsid assembly modulators or core protein allosteric modulators [CAMs (Zlotnick et al., 2015), CpAMs (Zlotnick et al., 2015)]. Their major mechanism has been described either to be the acceleration of capsid assembly kinetics, which promotes the formation of morphologically normal capsid structures, but results in a failure to package pgRNA, as observed for example for AT-130. Or induction of aberrant oversized Cp structures (Diab et al., 2018), sequestering capsids from their functions, as observed for heteroaryldihydropyrimidines (HAP). To avoid the confusion in the literature as to which mechanism of action is to be called class I vs. class II (Lahlali et al., 2018; Yang et al., 2019) we herein use a tentative new nomenclature whereby CAM-N refers to modulators causing normal and CAM-A to modulators causing abnormal capsid structures. CAMs interfere with several central steps in the viral life cycle. They have been shown to prevent nuclear transport of capsids blocking *de novo* formation of covalently closed circular (ccc) DNA (Nassal, 2015); they are active pan-genotypic, and active against nucleoside analog resistant virus mutants. Several CAMs of both classes are being evaluated in clinical trials (Durantel and Zoulim, 2016; Feng et al., 2018; Schinazi et al., 2018).

The capsid structure has been investigated by a range of structural-biology techniques. With the exception of a 3.3 Å X-ray structure (Wynne et al., 1999) of the N-terminal assembly domain, structures of the full-length capsid have been determined by cryo-electron microscopy (cryo-EM) (Crowther et al., 1994; Bottcher et al., 1997), the latest to date at 2.7 Å resolution (Böttcher and Nassal, 2018). The different cryo-EM structures have mostly been described as similar to the X-ray structure, although small differences have been attributed to the absence/presence of the CTD (Yu et al., 2013), the presence of RNA as opposed to DNA (Roseman et al., 2005), or to drug binding (Schlicksup et al., 2018). Importantly, while the CTD is present in the structures solved, it is flexible and has not a defined density (Zlotnick et al., 1997; Patel et al., 2017).

Eight structures of capsids with antiviral compounds bound have been determined (Bourne et al., 2006; Katen et al., 2013; Klumpp et al., 2015; Qiu et al., 2016; Venkatakrishnan et al., 2016; Zhou et al., 2017; Schlicksup et al., 2018). All characterized Cps carried mutations, and none contained the CTD. The most commonly used constructs were Cp150 carrying an unnatural C-terminal cysteine plus triple Cys to Ala mutations depleting the protein from all endogenous cysteine residues (3CA-Cp150C). These mutations maintain the symmetry used in cryo-EM reconstruction of capsid structures, as the covalent intra-capsid cross linking via the C-terminal cysteine counterbalances the drastic destabilizing effects of the investigated HAP1 CAM. The increased stability of the modified capsids enabled X-ray structures with a decent resolution of around 4 Å (Venkatakrishnan et al., 2016). A higher resolution (1.7 Å) structure was obtained by X-ray crystallography employing the Cp Y132A mutation (Qiu et al., 2016) that abrogates capsid formation. Instead, Y132A induces flat hexameric structures (trimers of dimers) which form excellent crystals but clearly do not reflect the structure of the assembled capsid which is important when considering CAM action (Schlicksup et al., 2018).

Overall it remains unclear, at a molecular level, whether assembly modulators act similarly on preassembled vs. nascent capsids. One approach to address this issue is to disassemble capsids into dimeric Cp subunits which then are incubated with the CAM under assembly-favoring conditions, e.g., high concentrations of salt (Schlicksup et al., 2018). Such non-physiological conditions could possibly interfere with assembly modulation. Studying assembly modulation instead directly at the exit from the ribosome, under conditions close to the cellular environment, is thus of high interest. This can principally be achieved using cell-free protein synthesis (CFPS). CFPS of the HBV capsid has been described early-on by Lingappa and coworkers (Lingappa et al., 1994) who produced viral capsids in wheat-germ extract cell-free system (WGE-CF). Their study was motivated by the question how capsid assembly is influenced, under near-physiological concentrations, by cellular proteins, the cytoplasmic environment, and organelles (Lingappa et al., 1994). Indeed, in cells, the concentration of capsid protein is relatively low (an estimate of the steady-state HBc concentration in stably transfected hepatoma cells established ca. 300 nM Ludgate et al., 2016). Another important point is that assembly and its modulation with purified protein differs from that in cells where capsid formation is linked to Cp translation (Lingappa et al., 2005) and occurs in the presence of chaperones. A rabbit reticulocyte lysate (RRL) cell-free system has been recently applied to study HBV capsid assembly under more physiological conditions; Cp is expressed with low concentrations and assembles under near-physiological conditions (Ludgate et al., 2016; Liu and Hu, 2018). However, this system generally does not yield quantities [about 250 ng per mL reaction (Ludgate et al., 2016)] sufficient for structural studies, notably by NMR.

While cell-free expression can provide a means to sample capsid modulation directly at the exit from the ribosome, the approach remains limited without a means to structurally analyze the products at atomic resolution. Solid-state NMR can study full-length, wild-type capsids simply as sediments resulting from ultracentrifugation (Goldbourt et al., 2007; Han et al., 2010; Andreas et al., 2016). Notably, NMR has low requirements on sample properties: they neither need to be crystalline, nor show symmetry, only local order. This allows comparisons of the NMR signals of a variety of preparations and forms, and to conclude about structural and dynamic differences. NMR can in principle provide spectral fingerprints relating to structural features for both normal and abnormal capsid induced by modulators, including for capsids carrying modifications like phosphorylation. As the necessary basis for further studies, we have recently assigned the NMR signals of the HBV capsid (Lecoq et al., 2018b), revealing residues which conformationally adapt to allow for the dimer-to-capsid transition. Also, we identified the residues of the core protein which form the hinges that accommodate formation of the quasi-equivalent five-fold and quasi-six-fold vertices in the capsid (Lecoq et al., 2018a).

The classical approach to solid-state NMR involving carbon-13 detection is difficult to apply to the milligram quantities CFPS can easily produce. The recent development of proton-detection techniques opens the way for such studies, as it reduces the necessary protein amount by almost two orders of magnitude, to submilligram quantities (Böckmann et al., 2015; Lecoq et al., 2019). We have recently shown that the duck hepatitis B virus (DHBV) subviral particles can auto-assemble in the cell-free system and be analyzed by NMR (David et al., 2018). We show here that this same system can be used to produce wild-type full-length Cp HBV capsids, and do so in amounts compatible with solid-state NMR structural investigations, including the recording of 3D spectra with sufficient resolution and sensitivity. We show that the phenotypes produced by CAM-N and CAM-A are similar to those produced using purified capsids from *E. coli*. Hence, WGE-CF synthesis of capsids combined with solid-state NMR provides a valuable tool to study the effects of capsid assembly modulation on proteins directly at the exit of the ribosome.

## Materials and Methods

### Plasmids

The genescorresponding either to the full-length core protein (Cp183) or to its truncated form Cp149 were cloned into the pEU-E01-MCS vector (CellFree Sciences, Japan) for WGE-CF expression. The plasmids were amplified in DH5α bacteria, and purified using a NucleoBond Xtra Maxi kit (Macherey-Nagel, France). An additional purification step was performed with a phenol/chloroform extraction to ensure the purity of the plasmid according to the recommendations of CellFree Sciences (Yokohama, Japan).

### mRNA Transcription

Transcription was performed according to Takai et al. (2010) in 1.5 mL Eppendorf tubes using 100 μg/mL plasmid, 2.5 mM NTP mix (Promega), 1 U/μL SP6 RNA Polymerase (CellFree Sciences), and 1U/μL RNase inhibitor (CellFree Sciences) in transcription buffer (CellFree Sciences) containing 80 mM Hepes-KOH pH 7.6, 16 mM magnesium acetate, 10 mM DTT and 2 mM spermidine. After incubation for 6 h at 37°C, mRNA was used directly for translation.

### Wheat Germ Cell-Free Protein Synthesis

Non-treated durum wheat seeds (Sud Céréales, France) were used to prepare home-made WGE as described in Fogeron et al. (2017), according to the protocol of Takai et al. (2010) with minor modifications. Translation was performed using the bilayer method as described in Takai et al. (2010), Fogeron et al. (2017) for small scale expression tests in the presence of compounds, or using the dialysis mode as described in David et al. (2018) for larger scale production followed by isolation on a sucrose density gradient. For the bilayer method, the bottom layer (20 μL) corresponding to the translation mixture contains per well 10 μL of mRNA, 10 μL of WGE, 40 ng/μL of creatine kinase and 6 mM of amino-acid mix (0.3 mM per amino acid, average concentration). The upper layer (200 μL) corresponding to the feeding buffer contains SUB-AMIX NA (CellFree Sciences; 30 mM Hepes-KOH pH 7.6, 100 mM potassium acetate, 2.7 mM magnesium acetate, 16 mM creatine phosphate, 0.4 mM spermidine, 1.2 mM ATP, 0.25 mM GTP, and 4 mM DTT), and 6 mM of amino acid mix (0.3 mM per amino acid, average concentration). For Cp183 expression in the presence of different compounds, 10 nmol of antiviral (dissolved in DMSO at a concentration of 10 mM) was added into 200 μL feeding buffer and translation was performed at 22°C for 16 h.

For large-scale production, dialysis cassettes with a volume of either 500 μL or 3 mL, depending on the production scale, and a MWCO of 10 kDa were used. The translation mixture contained ½ by volume of feeding buffer, 1/3 of mRNA, 1/6 of WGE, 40 ng/μL of creatine kinase, 0.3 mM of amino-acid mix. The feeding buffer (either 20 mL or 124 mL for a 500-μL or a 3-mL dialysis cassette, respectively) contains SUB-AMIX NA (CellFree Sciences) as described above, supplemented with 0.3 mM of amino-acid mix. The dialysis cassette containing the translation mix was soaked in the feeding buffer, and incubated for 16 h under shaking at 60 rpm, 22°C. A mix containing all twenty isotopically labeled amino acids (Cambridge Isotope Laboratory) was used for the production of ^13^C-^15^N-Cp183 for NMR studies in a 3 mL-translation reaction experiment.

### Isolation of the Capsids on a Sucrose Density Gradient

The total cell-free reaction mixture (CFS) was treated with 25,000 units/mL of benzonase for 30 min at room temperature before centrifugation at 20,000 g, 4°C for 30 min. The supernatant (SN) was loaded onto a discontinuous sucrose gradient with layers of 10, 20, 30, 40, 50, and 60% sucrose (w/v), each with a volume of 350 μL for a production in a 500-μL cassette. For the production of a ^13^C-^15^N-Cp183 sample in a 3-mL dialysis cassette, the supernatant (SN) was split into two fractions and loaded onto two sucrose gradients with layers of 10, 20, 30, 40, 50, and 60% sucrose (w/v), each with a volume of 1.5 mL. The gradients were centrifuged at 200,000 g, 4°C for 12 h. After centrifugation, the different sucrose fractions were harvested and analyzed by SDS-PAGE and Western blotting, as well as by electron microscopy after negative staining as described below.

### Capsids From *E. coli*

Cp183 capsids used as reference for negative stain EM with CAMs were obtained from BL21^*^-CodonPlus (DE3) cells using plasmid pRSF-T7-HBc183opt. Expression and purification were done as previously reported (Heger-Stevic et al., 2018a; Lecoq et al., 2018b). In brief, protein was expressed overnight after induction with 1 mM IPTG at 20°C, and cell lysate was separated with 10–60% sucrose gradient. Cp183 capsids were precipitated after the sucrose gradient by 40% saturation ammonium sulfate, and resuspended in final buffer (50 mM Tris pH 7.5, 5 mM DTT, 1 mM EDTA, 5% sucrose). The interaction between preformed capsids and compounds was performed with a molar ratio of Cp183 monomer: compound of 1:4, at 37°C for 2 h.

### Rotor Filling and NMR Data Acquisition

Four different Cp183 NMR samples were prepared: two from cell-free protein synthesis, one synthesized using ^13^C/^15^N, and the other one ^2^H/^13^C/^15^N amino acids, resulting in a protonated sample, and a deuterated, but 100% protonated on exchanging protons, as synthesis is carried out in H_2_O; and for reference two samples from *E. coli* expression, one deuterated and back exchanged on exchangeable sites, and one protonated (Heger-Stevic et al., 2018a; Lecoq et al., 2018b). NMR samples were filled into 0.7 mm rotors as sediment obtained by ultracentrifugation directly into the rotor (Böckmann et al., 2009) at 200,000 g for approximately 16 h at 4°C, yielding approximately 0.5 mg of sediment. As an internal chemical-shift reference, about 30 μL of saturated (0.3 M) 4,4-dimethyl-4-silapentane-1-sulfonic acid (DSS) was added to the protein solution before sedimentation.

On each of the samples a two-dimensional (2D) fingerprint hNH spectrum was recorded. On the protonated, uniformly ^13^C-^15^N labeled cell-free produced sample, an hCANH 3D (Penzel et al., 2015) was recorded in addition. All spectra were acquired on a wide-bore 850 MHz Bruker Avance III spectrometer with a 0.7 mm triple-resonance MAS probe (Bruker Biospin) operated at 100 kHz MAS. Magic angle and shim for this probe were set using a 0.7 mm rotor with glycine ethylester by optimizing the intensity and *J*-coupling based splitting of the CO resonance (Penzel et al., 2018). The sample was cooled with a BCU (Bruker Cooling Unit) gas flow of 400 l/h with a VT (Variable Temperature) set to 272 K, corresponding to a sample temperature of approximately 22°C, extrapolated from the water chemical shift in a ^1^H 1D (Gottlieb et al., 1997; Böckmann et al., 2009). Detailed acquisition parameters can be found in Supplementary Table 1.

### NMR Data Processing

TopSpin 4.0.3 (Bruker Biospin) was used for the data acquisition and processing. 2D hNH spectra were processed with 1,024 points in ^1^H dimension (corresponding to 12.9 ms of acquisition time) and zero filling was applied to, respectively, 4,096 points in ^1^H and 1,024 points in ^15^N dimension. The 3D hCANH was processed with zero filling to, respectively 2m048 points in ^1^H, 128 points in ^15^N, and 256 points in ^13^C dimensions. All spectra were apodized with a shifted sine-bell window function using SSB = 3.5 in TopSpin. Linear prediction to twice the recorded number of points was applied in the ^15^N dimension for 2D hNH spectra of the protonated capsids produced by CFPS, and the deuterated *E. coli* capsids, in order to reach a similar number of points as acquired for the other samples. Spectral analyses were performed using the CcpNmr Analysis package 2.4.2 (Stevens et al., 2011). The proton linewidths were obtained using the parabolic fit function integrated on CcpNmr on six isolated peaks in the hNH spectra. The errors given represent the standard deviations between the six values. Signal-to-noise ratio were calculated on the bulk signals from 1D hNH spectra recorded and processed with similar parameters and divided by the square root of the number of scans.

### SDS-PAGE and Western Blotting Analysis

The expression of Cp183 was assessed by 15% Coomassie blue stained SDS–PAGE and Western blotting as described in Fogeron et al. (2015). A polyclonal rabbit antiserum against the N-terminal domain of the HBV core protein (a-c149) was used to detect both Cp149 and Cp183 on blots.

### Negative Staining Electron Micrographs

Samples for electron microscopy were negatively stained as described in Lecoq et al. (2018b). Briefly, 5 μL of each fraction were loaded on a carbon-coated grid (EMS Microscopy) and incubated for 2 min at room temperature. Remaining liquid was drained using Whatman paper. Grids were negatively stained on a 50-μL drop of 2% phosphotungstic acid (pH = 7) for 2 min at room temperature and observed with a JEM-1,400 transmission electron microscope operating at 100 kV.

## Results

### Full-Length Cp183 but Not CTD-Less Cp149 Self-Assembles Upon Cell-Free Protein Synthesis

**Figure 1:**
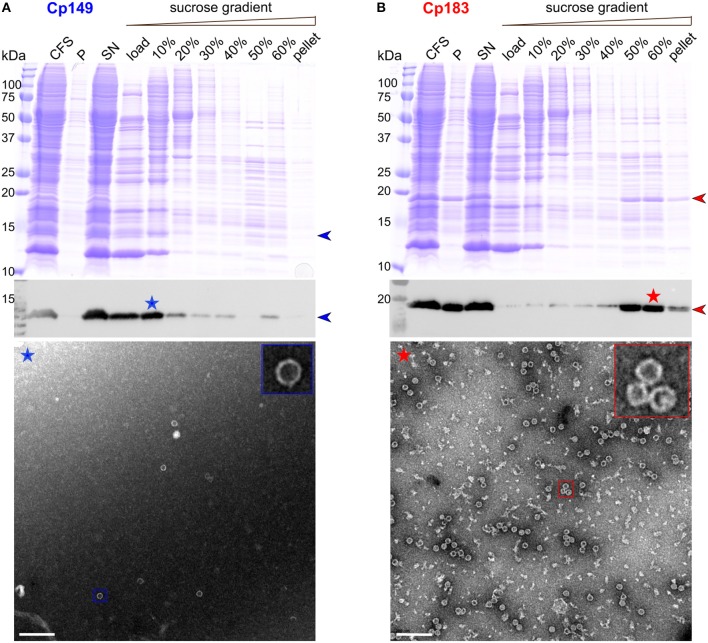
CFPS and sucrose gradient isolation of Cp149 **(A)** and Cp183 **(B)**. Shown are from top to bottom, Coomassie blue stained gels, western blots, and negative staining electron micrographs of protein-containing fractions. CFS: total cell-free reaction mixture; P and SN: pellet and supernatant obtained after centrifugation of the CFS at 20,000 g, 4°C for 30 min; 0–60%: fractions from the sucrose gradient. Scale bar = 200 nm.

CFPS of the core protein was performed for both Cp149 and Cp183. The protein was found mainly in the soluble fraction after centrifugation, as indicated in Western blots in Figures 1A,B. The protein band is partly visible in the total CFS fraction of the Coomassie blue gel. Enrichment via a sucrose gradient reveals that Cp149 stays mainly in the load and in the 10% sucrose fraction, indicating the protein remained in an unassembled, probably dimeric state. Accordingly, the electron micrograph of the 10% fraction (Figure 1A, blue asterisk), showed only very few capsids. In contrast, Cp183 sedimented largely into the 50 and 60% sucrose fractions (Figure 1B, red asterisk), as expected when capsids have been formed. EM inspection revealed numerous auto-assembled Cp183 capsids with a diameter of about 30 nm, as also observed for capsids assembled in *E. coli* (Gallina et al., 1989; Lecoq et al., 2018b).

Upon expression in *E. coli*, both Cp183 and the CTD-less Cp149 variant auto-assemble into capsids. Only full-length protein packages RNA, while Cp149 capsids remain empty (Birnbaum and Nassal, 1990). Both types of capsids can be isolated from bacteria by a set of purification steps (Heger-Stevic et al., 2018b; Lecoq et al., 2018b), with Cp149 giving particularly high yields (100 mg per liter of culture, compared to 20 mg/L for Cp183). The capsids can be disassembled using either urea (Cp149) or guanidinium chloride (Cp183) (Zlotnick et al., 1997; Porterfield et al., 2010). Reassembly is concentration dependent, and *in vitro* assembly of the full-length protein needs addition of nucleic acids which are non-sequence specifically packaged (Porterfield et al., 2010). Failure of Cp149 to assemble upon WGE-CF synthesis is likely due to the higher concentrations this protein needs for assembly, while the interaction between the positively-charged Cp183 CTD with the negatively-charged nucleic acids enables Cp183 assembly at concentrations as low as 5 nM (Klein et al., 2004). Failure of Cp149 to assemble has also been observed in rabbit reticulocyte extract (Ludgate et al., 2016).

### Milligram Amounts of ^13^C/^15^N Labeled Cp183 Capsids Can be Produced in Protonated and Deuterated Form

**Figure 2:**
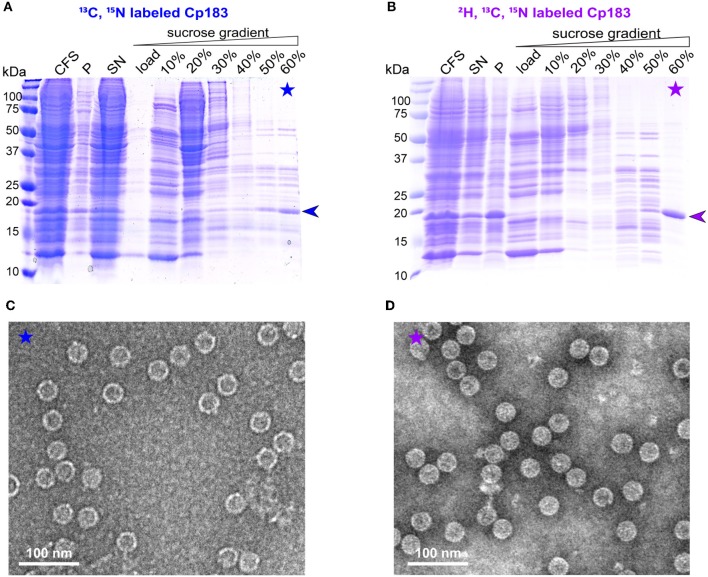
Analysis of CFPS and sucrose gradient isolation on 15% SDS-PAGE gels of **(A)** ^13^C, ^15^N and **(B)** ^2^H, ^13^C, ^15^N isotopically labeled dCp183. CFS: total cell-free reaction mixture; P and SN: pellet and supernatant obtained after centrifugation of the CFS at 20,000 g, 4°C for 30 min; 0–60%: fractions from the sucrose gradient. Negative staining electron micrographs display the ^13^C, ^15^N labeled **(C)** and ^2^H, ^13^C, ^15^N labeled **(D)** capsids from the 60% sucrose fractions. Scale bar = 100 nm.

For large-scale production (~1 milligram) needed for NMR sample preparation, CFPS was carried out in dialysis reactions, as described for the duck HBV envelope subviral particle synthesis (David et al., 2018). Either protonated or deuterated, HN protonated Cp183 was prepared, with the latter referred to in the following as dCp183. In the large-scale synthesis, more protein was found in the pellet compared to the small-scale synthesis, likely due to higher concentrations. On sucrose gradient isolation, migrated to the 60 % fraction (Figures 2A,B). The preparation using the deuterated amino acids shows higher purity, which might be due to a slightly different migration behavior of the deuterated protein in the sucrose gradient. EM inspection revealed abundant capsids in both preparations (Figures 2C,D).

### Cell-Free Synthesized Capsids Can be Analyzed by NMR

Conformational details can be revealed by NMR in so-called fingerprint spectra, which show either in two (2D) or three dimensions (3D) the typical signature of the protein preparation. Structural variations can be sensitively identified by comparing spectra recorded under different conditions, and analyzing the differences in the observed chemical shifts, i.e., the NMR frequencies (Williamson, 2013). An opportunity of the combination of CFPS and NMR is the fact that only the synthesized protein, which is the sole isotopically labeled protein, will be observed in the spectra. The use of a simple sucrose gradient concentration step thus might not produce perfectly pure protein; still, only the protein of interest will produce signal in the spectra. A possible drawback might lie in a loss of signal-to-noise ratio (SNR) in the spectra, since the NMR sample container (rotor) also might contain residual contaminating proteins (Figure 2A). It is thus important to establish whether protein samples prepared by CFPS are indeed compatible with the recording of 2D and in particular 3D spectra in a reasonable amount of time.

**Figure 3:**
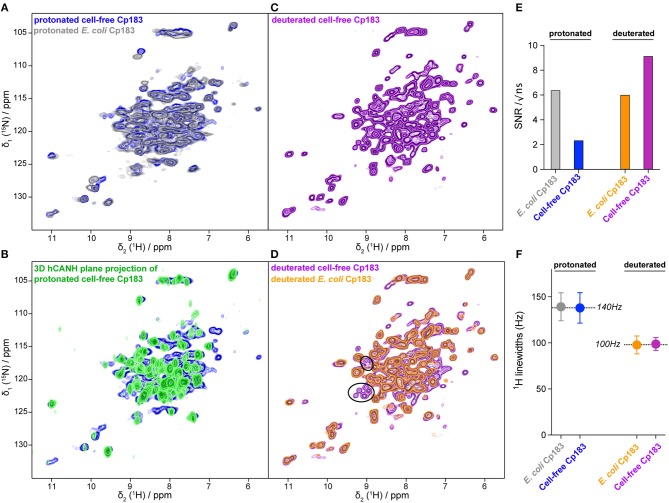
Comparisons of NMR spectra between the capsids from CFPS and capsids purified from *E. coli*. **(A)** Overlay of the 2D hHN spectra of the protonated Cp183 capsids from CFPS (in blue) and purified from *E. coli* (in gray); **(B)** overlay of the 2D planes from the 3D hCANH spectrum recorded on the protonated Cp183 CFPS capsids; **(C)** 2D hNH spectrum of the deuterated Cp183 CFPS capsids; **(D)** overlay of the 2D hNH spectra of the deuterated capsids from CFPS (in purple) and purified from *E. coli* (in orange), with resonances not observed in the *E. coli* sample highlighted as black circles. Spectra are shown individually in Figure S1. **(E)** Comparison of signal-to-noise ratios of the different samples; **(F)** comparison of proton linewidths in the different samples. The averages over the two protonated and the two deuterated samples are indicated.

The hNH 2D correlation spectrum recorded in 16 h on the protonated cell-free Cp183 displays a highly similar spectrum to the one recorded on the capsids purified from *E. coli* (Figure 3A and Figure S1) in 10 h. The NMR signal amplitude of the sample from CFPS is about 35% of the spectra obtained on the preparation from purified *E. coli* protein recorded under the same experimental conditions. As both rotors were full with protein sediment, this means that the contaminating unlabeled proteins from the WGE fill almost 2/3 of the rotor. A 3D hCANH spectrum was recorded on the sample in 4 days and 15 h, and an overlay of all 3D NH planes onto the 2D NH plane shows that most signals in the 2D hNH spectrum are also observed in the 3D (Figure 3B).

The 2D spectrum recorded on the deuterated sample is shown in Figures 3C,D. SNR is very favorable in this sample, since the deuterated protein surprisingly showed better purity (Figure 2B). The spectrum reveals narrower lines than the spectrum from the protonated sample, as also observed in model systems (Penzel et al., 2019) and, in particular, also in capsid preparations purified from *E. coli* (Lecoq et al., 2019): 140 Hz on average for the protonated vs. 100 Hz for the deuterated sample, as measured on six isolated resonances. The SNR and proton linewidths for the four samples are summarized in Figures 3E,F, respectively. It reveals that CFPS samples show a greater variability in sample amounts than the well-established *E. coli* samples; further experience is needed to evaluate parameters allowing reproducible sample preparation using CFPS. The proton linewidths are virtually similar between the two protonated and two deuterated samples, indicating that production by CFPS or *E. coli* expression does not make a difference with respect to linewidth and therefore conformational homogeneity.

Importantly, several peaks are present in the cell-free synthesized dCp183 which could not be observed in the deuterated sample purified from *E. coli*, as emphasized in Figure 3D. The origin of this observation lies in the incomplete back-exchange in *E. coli* produced samples. Indeed, when deuterated protein is expressed in *E. coli*, synthesis takes place in D_2_O, and exchange of deuterons to protons is achieved during the subsequent purification steps, carried out in H_2_O. Still, solvent-inaccessible deuterons can remain in the protein over long periods of time, and often denaturation/renaturation of the protein is applied to complete proton exchange important for NMR observation. However, this step can be very difficult for more complex proteins, and the present experiment highlights this interesting feature of CFPS, where the protein is synthesized from the beginning in H_2_O, and deuteration is achieved not via metabolism, but by addition of deuterated amino acids to the cell-free reaction. This results in fully protonated amide (and exchangeable sidechain) protons in the synthesized protein, which is essential for the recording of NMR spectra showing resonances for all amino acids.

### Capsids Can be Synthesized in the Presence of Antiviral Compounds

**Figure 4:**
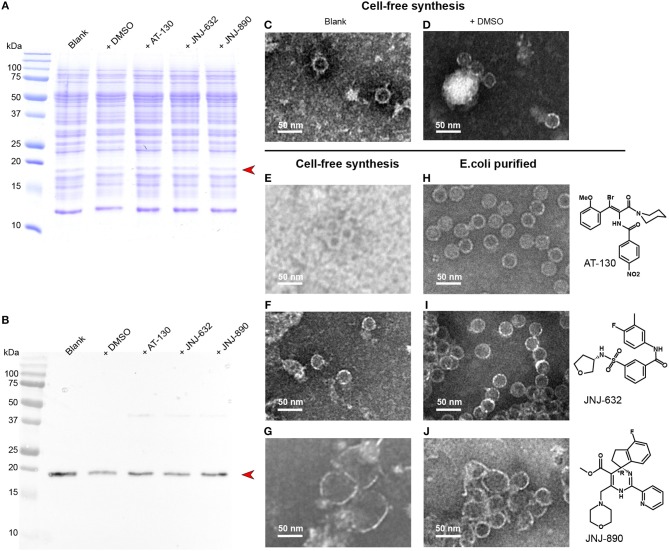
CFPS of Cp183 in presence of different compounds. **(A,B)** 15% SDS-PAGE **(A)** and Western blot **(B)** analysis on Cp183 protein produced in 220-μL CFPS system with 10 nmol drugs dissolved in 1 μL DMSO. The lanes of two controls, referred as *blank* and +*DMSO*, and productions with compounds are highlighted on the top. Electron microscopy (negative staining) analysis of cell-free products of the *blank* **(C)**, +*DMSO* **(D)**, +*AT-130* **(E)**, +*JNJ-632* **(F)**, +*JNJ-890* **(G)**, as well as capsids from *E.coli* incubated with AT-130 **(H)**, JNJ-632 **(I)**, JNJ-890 **(J)**, are shown on the right. Scale bar = 50 nm.

CFPS proceeds in an open system, and a variety of substances can be added to the reaction mixture. We added different capsid assembly modulators to the reaction, in order to analyze whether this produces comparable phenotypes to those observed on capsids purified from *E. coli*. Figure 4A shows the Coomassie blue stained gels of the cell-free solutions without compounds, in the presence of DMSO used for solubilization of the antiviral, and in presence of AT-130, JNJ-623 (CAM-N), and JNJ-890 (CAM-A). The corresponding Western blots are shown in Figure 4B. None of the compounds inhibited protein synthesis. We analyzed the total cell-free solutions, without any concentration or purification, under the electron microscope, and compared the observed capsids as shown in Figures 4C–G with the ones obtained from addition of compounds to capsids purified from *E. coli*, shown in Figures 4H–J. One can see in the micrographs that the resulting objects closely resemble those obtained by addition to preformed capsids: DMSO vehicle and CAM-Ns produced no visible effect, whereas CAM-As showed the typical disruption of capsids also reported in the literature (Berke et al., 2017; Lahlali et al., 2018). Notably, the presence of AT-130 lead to poorer contrast in the EM micrographs of both preparations.

## Conclusions

We have synthesized HBV viral capsids in a eukaryotic wheat germ cell-free system in sufficient amounts for structural analyses, including by solid-state NMR. We have shown that the full-length Cp183 protein auto-assembles in the cell-free system to form icosahedral capsids virtually identical to those obtained upon bacterial expression. This finding opens the possibility to produce isotope labeled samples, both in protonated and deuterated forms, for advanced proton-detected NMR experiments. The spectra recorded on the samples showed sufficient signal-to-noise to analyze 2D and 3D spectral fingerprints and thus conformational changes. Importantly, this enables investigations of capsid interactions directly on synthesis with assembly modulators, other natural compounds such as lipids, or chaperones and enzymes that might be relevant *in vivo*. We demonstrated this at the example of three capsid assembly modulators from different chemical classes, which induced similar structural changes in capsids synthesized and assembled in presence of the compounds and in preformed capsids isolated from *E. coli*. Hence the influence of small molecules on the capsid can now also be assessed on assembly after exit from the ribosome, on the relevant full-length protein, without extensive purification steps, and in the presence of nucleic acids.

## Data Availability

The datasets generated for this study are available on request to the corresponding author.

## Author Contributions

SW, M-LF, and MD carried out protein syntheses and analyses, and generated NMR samples. MS, SP, and LL conducted the NMR experiments. DB and JB provided antiviral compounds, and contributed expert insight to CAMs. MN designed the plasmid and established bacterial expression/purification protocols, and contributed expert insight to HBV. M-LF, LL, BM, and AB designed and supervised the study, and wrote the manuscript. All authors contributed to the manuscript and approved the submitted version.

### Conflict of Interest Statement

The authors declare that the research was conducted in the absence of any commercial or financial relationships that could be construed as a potential conflict of interest.

## References

[B1] Andreas L. B. Jaudzems K. Stanek J. Lalli D. Bertarello A. Le Marchand T.. (2016). Structure of fully protonated proteins by proton-detected magic-angle spinning NMR. Proc. Natl. Acad. Sci. U.S.A. 113, 9187–9192. 10.1073/pnas.1602248113 27489348 PMC4995937

[B2] Bartenschlager R. Junker-Niepmann M. Schaller H. (1990). The P gene product of hepatitis B virus is required as a structural component for genomic RNA encapsidation. J. Virol. 64, 5324–5332. 2214019 10.1128/jvi.64.11.5324-5332.1990 PMC248581

[B3] Berke J. M. Dehertogh P. Vergauwen K. Van Damme E. Mostmans W. Vandyck K.. (2017). Capsid assembly modulators have a dual mechanism of action in primary human hepatocytes infected with hepatitis B virus. Antimicrob. Agents Chemother. 61:e00560–17. 10.1128/AAC.00560-17 28584155 PMC5527576

[B4] Birnbaum F. Nassal M. (1990). Hepatitis B virus nucleocapsid assembly: primary structure requirements in the core protein. J. Virol. 64, 3319–3330. 2191149 10.1128/jvi.64.7.3319-3330.1990 PMC249568

[B5] Blondot M.-L. Bruss V. Kann M. (2016). Intracellular transport and egress of hepatitis B virus. J. Hepatol. 64, S49–S59. 10.1016/j.jhep.2016.02.008 27084037

[B6] Böckmann A. Ernst M. Meier B. H. (2015). Spinning proteins, the faster, the better? J. Biomol. NMR. 253, 71–79. 10.1016/j.jmr.2015.01.012 25797006

[B7] Böckmann A. Gardiennet C. Verel R. Hunkeler A. Loquet A. Pintacuda G.. (2009). Characterization of different water pools in solid-state NMR protein samples. J. Biomol. NMR 45, 319–327. 10.1007/s10858-009-9374-3 19779834

[B8] Böttcher B. Nassal M. (2018). Structure of mutant hepatitis B core protein capsids with premature secretion phenotype. J. Mol. Biol. 430, 4941–4954. 10.1016/j.jmb.2018.10.018 30539760

[B9] Bottcher B. Wynne S. A. Crowther R. A. (1997). Determination of the fold of the core protein of hepatitis B virus by electron cryomicroscopy. Nature 386, 88–91. 10.1038/386088a0 9052786

[B10] Bourne C. R. Finn M. G. Zlotnick A. (2006). Global structural changes in hepatitis B virus capsids induced by the assembly effector HAP1. J. Virol. 80, 11055–11061. 10.1128/JVI.00933-06 16943288 PMC1642186

[B11] Crowther R. A. Kiselev N. A. Bottcher B. Berriman J. A. Borisova G. P. Ose V.. (1994). Three-dimensional structure of hepatitis B virus core particles determined by electron cryomicroscopy. Cell 77, 943–950. 10.1016/0092-8674(94)90142-2 8004680

[B12] David G. Fogeron M.-L. Schledorn M. Montserret R. Haselmann U. Penzel S.. (2018). Structural studies of self-assembled subviral particles: combining cell-free expression with 110 kHz MAS NMR spectroscopy. Angew. Chem. Int. Ed. 57, 4787–4791. 10.1002/anie.201712091 29457857

[B13] Diab A. Foca A. Zoulim F. Durantel D. Andrisani O. (2018). The diverse functions of the hepatitis B core/capsid protein (HBc) in the viral life cycle: Implications for the development of HBc-targeting antivirals. Antiviral Res. 149, 211–220. 10.1016/j.antiviral.2017.11.015 29183719 PMC5757518

[B14] Durantel D. Zoulim F. (2016). New antiviral targets for innovative treatment concepts for hepatitis B virus and hepatitis delta virus. J. Hepatol. 64, S117–S131. 10.1016/j.jhep.2016.02.016 27084032

[B15] Feng S. Gao L. Han X. Hu T. Hu Y. Liu H.. (2018). Discovery of small molecule therapeutics for treatment of chronic HBV infection. ACS Infect. Dis. 4, 257–277. 10.1021/acsinfecdis.7b00144 29369612

[B16] Fogeron M.-L. Badillo A. Jirasko V. Gouttenoire J. Paul D. Lancien L.. (2015). Wheat germ cell-free expression: two detergents with a low critical micelle concentration allow for production of soluble HCV membrane proteins. Protein Expr. Purif. 105, 39–46. 10.1016/j.pep.2014.10.003 25306874

[B17] Fogeron M.-L. Badillo A. Penin F. Böckmann A. (2017). Wheat germ cell-free overexpression for the production of membrane proteins. Methods Mol. Biol. 1635, 91–108. 10.1007/978-1-4939-7151-0_5 28755365

[B18] Gallina A. Bonelli F. Zentilin L. Rindi G. Muttini M. Milanesi G. (1989). A recombinant hepatitis B core antigen polypeptide with the protamine-like domain deleted self-assembles into capsid particles but fails to bind nucleic acids. J. Virol. 63, 4645–4652. 2677399 10.1128/jvi.63.11.4645-4652.1989 PMC251098

[B19] Gazina E. V. Fielding J. E. Lin B. Anderson D. A. (2000). Core protein phosphorylation modulates pregenomic RNA encapsidation to different extents in human and duck hepatitis B viruses. J. Virol. 74, 4721–4728. 10.1128/JVI.74.10.4721-4728.2000 10775610 PMC111994

[B20] Goldbourt A. Gross B. J. Day L. A. McDermott A. E. (2007). Filamentous phage studied by magic-angle spinning NMR: resonance assignment and secondary structure of the coat protein in Pf1. J. Am. Chem. Soc. 129, 2338–2344. 10.1021/ja066928u 17279748

[B21] Gottlieb H. E. Kotlyar V. Nudelman A. (1997). NMR chemical shifts of common laboratory solvents as trace impurities. J. Org. Chem. 62, 7512–7515. 10.1021/jo971176v 11671879

[B22] Han Y. Ahn J. Concel J. Byeon I.-J. L. Gronenborn A. M. Yang J.. (2010). Solid-state NMR studies of HIV-1 capsid protein assemblies. JACS 132, 1976–1987. 10.1021/ja908687k 20092249 PMC2829833

[B23] Heger-Stevic J. Kolb P. Walker A. Nassal M. (2018a). Displaying whole-chain proteins on hepatitis B virus capsid-like particles. Methods Mol. Biol. 1776, 503–531. 10.1007/978-1-4939-7808-3_33 29869263

[B24] Heger-Stevic J. Zimmermann P. Lecoq L. Böttcher B. Nassal M. (2018b). Hepatitis B virus core protein phosphorylation: identification of the SRPK1 target sites and impact of their occupancy on RNA binding and capsid structure. PLoS Pathog. 14:e1007488. 10.1371/journal.ppat.1007488 30566530 PMC6317823

[B25] Kann M. Gerlich W. H. (1994). Effect of core protein phosphorylation by protein kinase C on encapsidation of RNA within core particles of hepatitis B virus. J. Virol. 68, 7993–8000. 7966589 10.1128/jvi.68.12.7993-8000.1994 PMC237262

[B26] Katen S. P. Tan Z. Chirapu S. R. Finn M. G. Zlotnick A. (2013). Assembly-directed antivirals differentially bind quasiequivalent pockets to modify hepatitis B virus capsid tertiary and quaternary structure. Structure 21, 1406–1416. 10.1016/j.str.2013.06.013 23871485 PMC3756818

[B27] Klein K. C. Polyak S. J. Lingappa J. R. (2004). Unique features of hepatitis C virus capsid formation revealed by de novo cell-free assembly. J. Virol. 78, 9257–9269. 10.1128/JVI.78.17.9257-9269.2004 15308720 PMC506955

[B28] Klumpp K. Lam A. M. Lukacs C. Vogel R. Ren S. Espiritu C.. (2015). High-resolution crystal structure of a hepatitis B virus replication inhibitor bound to the viral core protein. Proc. Natl. Acad. Sci. U.S.A. 112, 15196–15201. 10.1073/pnas.1513803112 26598693 PMC4679053

[B29] Lahlali T. Berke J. M. Vergauwen K. Foca A. Vandyck K. Pauwels F.. (2018). Novel potent capsid assembly modulators regulate multiple steps of the hepatitis B virus life cycle. Antimicrob. Agents Chemother. 62, 672–615. 10.1128/AAC.00835-18 30012770 PMC6153789

[B30] Lecoq L. Schledorn M. Wang S. Smith-Penzel S. Malär A. A. Callon M. (2019). 100 kHz MAS proton-detected NMR spectroscopy of hepatitis B virus capsids. Front. Mol. Biosci. 6:80 10.3389/fmolb.2019.00058 PMC6668038 31396521

[B31] Lecoq L. Wang S. Wiegand T. Bressanelli S. Nassal M. Meier B. H.. (2018a). Localizing conformational hinges by NMR: where do hepatitis B virus core proteins adapt for capsid assembly? Chemphyschem. 19, 1336–1340. 10.1002/cphc.201800211 29542854

[B32] Lecoq L. Wang S. Wiegand T. Bressanelli S. Nassal M. Meier B. H.. (2018b). Solid-state [^13^C-^15^N] NMR resonance assignment of hepatitis B virus core protein. Biomol. NMR Assign. 12, 205–214. 10.1007/s12104-018-9810-y 29450824

[B33] Lingappa J. R. Martin R. L. Wong M. L. Ganem D. Welch W. J. Lingappa V. R. (1994). A eukaryotic cytosolic chaperonin is associated with a high molecular weight intermediate in the assembly of hepatitis B virus capsid, a multimeric particle. J. Cell Biol. 125, 99–111. 10.1083/jcb.125.1.99 7908022 PMC2120005

[B34] Lingappa J. R. Newman M. A. Klein K. C. Dooher J. E. (2005). Comparing capsid assembly of primate lentiviruses and hepatitis B virus using cell-free systems. Virology 333, 114–123. 10.1016/j.virol.2004.12.024 15708597

[B35] Liu K. Hu J. (2018). Host-regulated hepatitis B virus capsid assembly in a mammalian cell-free system. Bio. Protoc. 8:e2813. 10.21769/BioProtoc.2813 29770355 PMC5951416

[B36] Ludgate L. Liu K. Luckenbaugh L. Streck N. Eng S. Voitenleitner C.. (2016). Cell-free hepatitis B virus capsid assembly dependent on the core protein C-terminal domain and regulated by phosphorylation. J. Virol. 90, 5830–5844. 10.1128/JVI.00394-16 27076641 PMC4886785

[B37] Nassal M. (1992). The arginine-rich domain of the hepatitis B virus core protein is required for pregenome encapsidation and productive viral positive-strand DNA synthesis but not for virus assembly. J. Virol. 66, 4107–4116. 1602535 10.1128/jvi.66.7.4107-4116.1992 PMC241213

[B38] Nassal M. (2008). Hepatitis B viruses: reverse transcription a different way. Virus Res. 134, 235–249. 10.1016/j.virusres.2007.12.024 18339439

[B39] Nassal M. (2015). HBV cccDNA: viral persistence reservoir and key obstacle for a cure of chronic hepatitis B. Gut 64, 1972–1984. 10.1136/gutjnl-2015-309809 26048673

[B40] Nijampatnam B. Liotta D. C. (2019). Recent advances in the development of HBV capsid assembly modulators. Curr. Opin. Chem. Biol. 50, 73–79. 10.1016/j.cbpa.2019.02.009 30952041

[B41] Patel N. White S. J. Thompson R. F. Bingham R. Weiß E. U. Maskell D. P.. (2017). HBV RNA pre-genome encodes specific motifs that mediate interactions with the viral core protein that promote nucleocapsid assembly. Nat. Microbiol. 2:17098. 10.1038/nmicrobiol.2017.98 28628133 PMC5495169

[B42] Penzel S. Oss A. Org M.-L. Samoson A. Böckmann A. Ernst M. (2019). Spinning faster: protein NMR at MAS frequencies up to 126 kHz. J. Biomol. NMR 128, 12620–12611. 10.1007/s10858-018-0219-9 PMC6441448 30680507

[B43] Penzel S. Smith A. A. Agarwal V. Hunkeler A. Org M.-L. Samoson A.. (2015). Protein resonance assignment at MAS frequencies approaching 100 kHz: a quantitative comparison of J-coupling and dipolar-coupling-based transfer methods. J. Biomol. NMR 63, 165–186. 10.1007/s10858-015-9975-y 26267840

[B44] Penzel S. Smith A. A. Ernst M. Meier B. H. (2018). Setting the magic angle for fast magic-angle spinning probes. J. Magn. Reson. 293, 115–122. 10.1016/j.jmr.2018.06.002 29929181

[B45] Porterfield J. Z. Dhason M. S. Loeb D. D. Nassal M. Stray S. J. Zlotnick A. (2010). Full-length hepatitis B virus core protein packages viral and heterologous RNA with similarly high levels of cooperativity. J. Virol. 84, 7174–7184. 10.1128/JVI.00586-10 20427522 PMC2898219

[B46] Qiu Z. Lin X. Zhou M. Liu Y. Zhu W. Chen W.. (2016). Design and synthesis of orally bioavailable 4-methyl heteroaryldihydropyrimidine based hepatitis B virus (HBV) capsid inhibitors. J. Med. Chem. 59, 7651–7666. 10.1021/acs.jmedchem.6b00879 27458651

[B47] Roseman A. M. Berriman J. A. Wynne S. A. Butler P. J. G. Crowther R. A. (2005). A structural model for maturation of the hepatitis B virus core. Proc. Natl. Acad. Sci. U.S.A. 102, 15821–15826. 10.1073/pnas.0504874102 16247012 PMC1276056

[B48] Schinazi R. F. Ehteshami M. Bassit L. Asselah T. (2018). Towards HBV curative therapies. Liver Int. 38, 102–114. 10.1111/liv.13656 29427479 PMC6481632

[B49] Schlicksup C. J. Wang J. C.-Y. Francis S. Venkatakrishnan B. Turner W. W. VanNieuwenhze M.. (2018). Hepatitis B virus core protein allosteric modulators can distort and disrupt intact capsids. Elife 7:13046. 10.7554/eLife.31473 29377794 PMC5788503

[B50] Seeger C. Mason W. S. (2015). Molecular biology of hepatitis B virus infection. Virology 479–480, 672–686. 10.1016/j.virol.2015.02.031 25759099 PMC4424072

[B51] Stevens T. J. Fogh R. H. Boucher W. Higman V. A. Eisenmenger F. Bardiaux B.. (2011). A software framework for analysing solid-state MAS NMR data. J. Biomol. NMR 51, 437–447. 10.1007/s10858-011-9569-2 21953355 PMC3222832

[B52] Takai K. Sawasaki T. Endo Y. (2010). Practical cell-free protein synthesis system using purified wheat embryos. Nat. Protoc. 5, 227–238. 10.1038/nprot.2009.207 20134421

[B53] Venkatakrishnan B. Katen S. P. Francis S. Chirapu S. Finn M. G. Zlotnick A. (2016). Hepatitis B virus capsids have diverse structural responses to small-molecule ligands bound to the heteroaryldihydropyrimidine pocket. J. Virol. 90, 3994–4004. 10.1128/JVI.03058-15 26842475 PMC4810570

[B54] Williamson M. P. (2013). Using chemical shift perturbation to characterise ligand binding. Progr. NMR Spectr. 73, 1–16. 10.1016/j.pnmrs.2013.02.001 23962882

[B55] Wynne S. A. Crowther R. A. Leslie A. G. W. (1999). The crystal structure of the human hepatitis B virus capsid. Mol. Cell 3, 771–780. 10.1016/S1097-2765(01)80009-5 10394365

[B56] Yang L. Liu F. Tong X. Hoffmann D Zuo J. Lu M. (2019). Treatment of chronic hepatitis B virus infection using small molecule modulators of nucleocapsid assembly: recent advances and perspectives. ACS Infect. Dis. 5, 713–724. 10.1021/acsinfecdis.8b00337 30896149

[B57] Yang L. Lu M. (2018). Small molecule inhibitors of hepatitis B virus nucleocapsid assembly: a new approach to treat chronic HBV infection. Curr. Med. Chem. 25, 802–813. 10.2174/0929867324666170704121800 28675991

[B58] Yu X. Jin L. Jih J. Shih C. Zhou Z. H. (2013). 3.5Å cryoEM structure of hepatitis B virus core assembled from full-length core protein. PLoS ONE 8:e69729. 10.1371/journal.pone.0069729 24039702 PMC3765168

[B59] Zhou Z. Hu T. Zhou X. Wildum S. Garcia-Alcalde F. Xu Z.. (2017). Heteroaryldihydropyrimidine (HAP) and sulfamoylbenzamide (SBA) inhibit hepatitis B virus replication by different molecular mechanisms. Sci. Rep. 7:42374. 10.1038/srep42374 28205569 PMC5304331

[B60] Zlotnick A. Cheng N. Stahl S. J. Conway J. F. Steven A. C. Wingfield P. T. (1997). Localization of the C terminus of the assembly domain of hepatitis B virus capsid protein: implications for morphogenesis and organization of encapsidated RNA. Proc. Natl. Acad. Sci. U.S.A. 94, 9556–9561. 10.1073/pnas.94.18.9556 9275161 PMC23216

[B61] Zlotnick A. Venkatakrishnan B. Tan Z. Lewellyn E. Turner W. Francis S. (2015). Core protein: a pleiotropic keystone in the HBV lifecycle. Antiviral Res. 121, 82–93. 10.1016/j.antiviral.2015.06.020 26129969 PMC4537649

