# Supplementary material for: Combining Cell-Free Protein Synthesis and NMR Into a Tool to Study Capsid Assembly Modulation
Source: Front Mol Biosci. 2019 Aug 8;6:67. doi: 10.3389/fmolb.2019.00067 (PMC6694763; doi:10.3389/fmolb.2019.00067)
Supplement: Supplementary file 1 [file Data_Sheet_1.PDF]

# *Supplementary Material*

## Combining cell-free protein synthesis and NMR into a tool to study capsid assembly modulation

Shishan Wang[1], Marie-Laure Fogeron[1], Maarten Schledorn[2], Marie Dujardin[1], Susanne Penzel[3], Dara Burdette[3], Jan Martin Berke[4], Michael Nassal[5], Lauriane Lecoq[1], Beat H. Meier[2*] & Anja Böckmann[1*]

[1]*Institut de Biologie et Chimie des Protéines, MMSB, Labex Ecofect, UMR 5086 CNRS, Université de Lyon, 7 passage du Vercors, 69367 Lyon, France*

[2]*Physical Chemistry, ETH Zurich, 8093 Zurich, Switzerland*

[3]*Gilead Sciences, 333 Lakeside Dr., Foster City, CA 94404*

[4]*Janssen Pharmaceutica N.V., Turnhoutseweg 30, 2340 Beerse, Belgium*

[5]*University Hospital Freiburg, Internal Medicine II / Molecular Biology, Hugstetter Str. 55, D-79106 Freiburg, Germany*

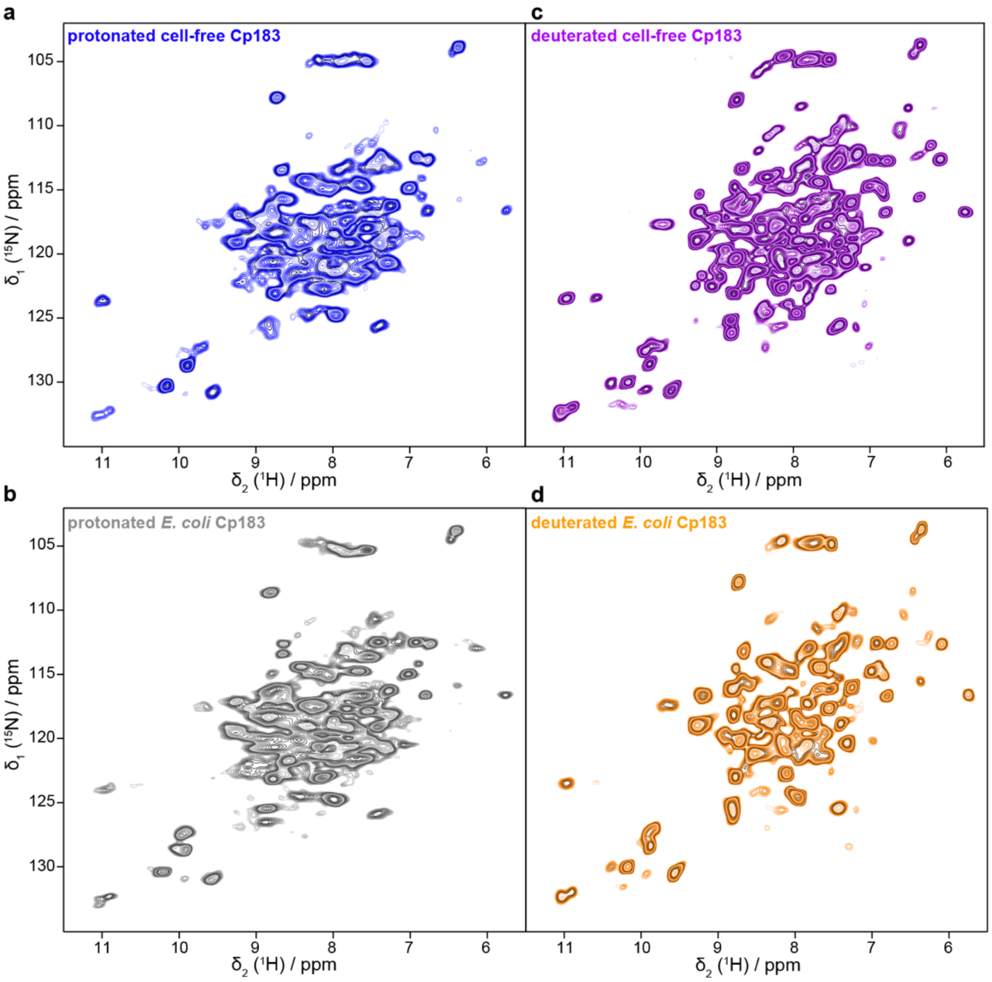


**Figure S1.** Individual spectra from Figure 3.

**Supplementary Table 1:** NMR experimental parameters for the presented data acquired on Cp183 capsids.

| **Sample production** | ***E. coli*** | ***E. coli*** | **Cell-free** | **Cell-free** | |
|---|---|---|---|---|---|
| **Sample labeling** | **$^{2}H$ $^{13}C$ $^{15}N$** | **$^{1}H$ $^{13}C$ $^{15}N$** | **$^{2}H$ $^{13}C$ $^{15}N$** | **$^{1}H$ $^{13}C$ $^{15}N$** | |
| **Experiment** | **hNH 2D** | **hNH 2D** | **hNH 2D** | **hNH 2D** | **hCANH 3D** |
| MAS frequency / kHz | 100 | 100 | 100 | 100 | 100 |
| Field / T | 20 | 20 | 20 | 20 | 20 |
| t1 increments | 284 | 774 | 620 | 330 | 104 |
| Sweep width (t1) / ppm | 180 | 180 | 180 | 180 | 36 |
| Acquisition time (t1) / ms | 9.2 | 25 | 20 | 11 | 6.8 |
| t2 increments | 5550 | 5550 | 2048 | 2048 | 44 |
| Sweep width (t2) / ppm | 47 | 47 | 47 | 40 | 34 |
| Acquisition time (t2) / ms | 70 | 70 | 26 | 30 | 7.5 |
| t3 increments | - | - | - | - | 2048 |
| Sweep width (t3) / ppm | - | - | - | - | 47 |
| Acquisition time (t3) / ms | - | - | - | - | 26 |
| Proton decoupling (swfTPPM) / kHz | 11 | 12 | 10 | 10 | 10 |
| Nitrogen decoupling (WALTZ64) / kHz | 6 | 6 | 5 | 5 | 5 |
| Carbon decoupling (WALTZ64) / kHz | - | - | - | - | 5 |
| Water suppression (120 ms MISS.) / kHz | 22 | 24 | 20 | 20 | 20 |
| Interscan delay / s | 2.0 | 1.2 | 2.0 | 1.2 | 1.2 |
| Number of scans | 32 | 32 | 64 | 128 | 64 |
| Measurement time / dd:hh:mm | 00:15:34 | 00:09:50 | 00:16:41 | 00:15:59 | 04:14:32 |
| Carrier $^{1}H$ / ppm | 4.8 | 4.8 | 4.8 | 4.8 | 4.8 |
| Carrier $^{15}N$ / ppm | 117.5 | 117.5 | 117.5 | 117.5 | 117.5 |
| **Transfer I** | **HN CP** | **HN CP** | **HN CP** | **HN CP** | **HC CP** |
| $^{1}H$ field / kHz | 72 | 77 | 78 | 77 | 82 |
| X field / kHz | 17 | 16 | 15 | 15 | 15 |
| Shape | Tangent $^{1}H$ | Tangent $^{1}H$ | Tangent $^{1}H$ | Tangent $^{1}H$ | Tangent $^{1}H$ |
| Carrier $^{13}C$ / ppm | - | - | - | - | 56 |
| Time / ms | 1.0 | 1.0 | 1.8 | 1.6 | 0.6 |
| **Transfer II** | **NH CP** | **NH CP** | **NH CP** | **NH CP** | **CN CP** |
| $^{1}H$ field / kHz | 72 | 77 | 78 | 77 | - |
| $^{13}C$ field / kHz | - | - | - | - | 63 |
| $^{15}N$ field / kHz | 17 | 16 | 15 | 15 | 35 |
| Shape | Tangent $^{1}H$ | Tangent $^{1}H$ | Tangent $^{1}H$ | Tangent $^{1}H$ | Tangent $^{13}C$ |
| Carrier $^{13}C$ / ppm | - | - | - | - | 56 |
| Time / ms | 1.0 | 1.0 | 2.4 | 1.6 | 20.0 |
| **Transfer III** | **-** | **-** | **-** | **-** | **NH CP** |
| $^{1}H$ field / kHz | - | - | - | - | 78 |
| $^{15}N$ field / kHz | - | - | - | - | 15 |
| Shape | - | - | - | - | Tangent $^{1}H$ |
| Time / ms | - | - | - | - | 1.4 |
